# TYROBP, TLR4 and ITGAM regulated macrophages polarization and immune checkpoints expression in osteosarcoma

**DOI:** 10.1038/s41598-021-98637-x

**Published:** 2021-09-29

**Authors:** Tuo Liang, Jiarui Chen, GuoYong Xu, Zide Zhang, Jiang Xue, Haopeng Zeng, Jie Jiang, Tianyou Chen, Zhaojie Qin, Hao Li, Zhen Ye, Yunfeng Nie, Chong Liu, Xinli Zhan

**Affiliations:** 1 Department of Spine and Osteopathy Ward, The First Affiliated Hospital of Guangxi Medical University, No. 6 Shuangyong Road, Nanning, Guangxi 530021 People’s Republic of China; 2 Guangxi Medical University, No.22 Shuangyong Road, Nanning, Guangxi People’s Republic of China

**Keywords:** Biomarkers, Diagnostics, Drug regulation, Sarcoma, Tumour immunology

## Abstract

We established a relationship among the immune-related genes, tumor-infiltrating immune cells (TIICs), and immune checkpoints in patients with osteosarcoma. The gene expression data for osteosarcoma were downloaded from UCSC Xena and GEO database. Immune-related differentially expressed genes (DEGs) were detected to calculate the risk score. “Estimate” was used for immune infiltrating estimation and “xCell” was used to obtain 64 immune cell subtypes. Furthermore, the relationship among the risk scores, immune cell subtypes, and immune checkpoints was evaluated. The three immune-related genes (TYROBP, TLR4, and ITGAM) were selected to establish a risk scoring system based on their integrated prognostic relevance. The GSEA results for the Hallmark and KEGG pathways revealed that the low-risk score group exhibited the most gene sets that were related to immune-related pathways. The risk score significantly correlated with the xCell score of macrophages, M1 macrophages, and M2 macrophages, which significantly affected the prognosis of osteosarcoma. Thus, patients with low-risk scores showed better results with the immune checkpoints inhibitor therapy. A three immune-related, gene-based risk model can regulate macrophage activation and predict the treatment outcomes the survival rate in osteosarcoma.

## Introduction

Osteosarcoma (OS) is a common form of high-graded primary malignant bone neoplasm in children and adolescents^1,2^. Statistical data reveal that incidence of OS is continuously growing by approximately 1.4% annually^3^. The two most commonly used clinical treatment methods for OS include systemic chemotherapy and local control surgery^4^. Despite several intensive efforts for a better prognosis, the high recurrence rate and early lung metastatic results in a poor prognosis poor for patients with OS^5,6^. Therefore, it is important to explore the carcinogenesis and therapeutics of OS.

An increasingly number of studies have demonstrated the significance of the tumor immune microenvironment in tumor progression^7,8^. TIICs in the tumor microenvironment (TME) influenced the tumor development and progression, which could serve as a potential marker for predicting the prognosis^9^. In addition, studies report that the expression of immune checkpoints such as PD-1, PD-L1, and CTLA-4 is associated with OS immune tolerance^10^. Immune checkpoint inhibitors, which restore the immune function of T cells and kill tumor cells, have been used to alleviate the immunosuppressive state of the TME in solid malignancies. Recently, scholars have constantly discussed the immune-related genes in TME, which may regulate TIICs and immune checkpoints. Therefore, it becomes important to enhance the knowledge about the TME, which can later help us identify novel immune therapy biomarkers for OS.

In this study, we constructed a risk model based on the TCGA cohort and validated it using the GSE21257 cohort. The three immune-related genes (TYROBP, TLR4 and ITGAM), the basis for the risk model, were strongly associated with macrophage polarization in the TME. Furthermore, the risk score was negatively correlated with the PD-1, PD-L1, and CTLA-4 immune checkpoint proteins that affect the survival rate and efficacy of the blockade of the immune checkpoint. Therefore, our risk model can predict the efficacy of immune checkpoints blockade in OS.

## Results

### EMT-related DEGs were mainly involved in inflammation response

**Figure 1:**
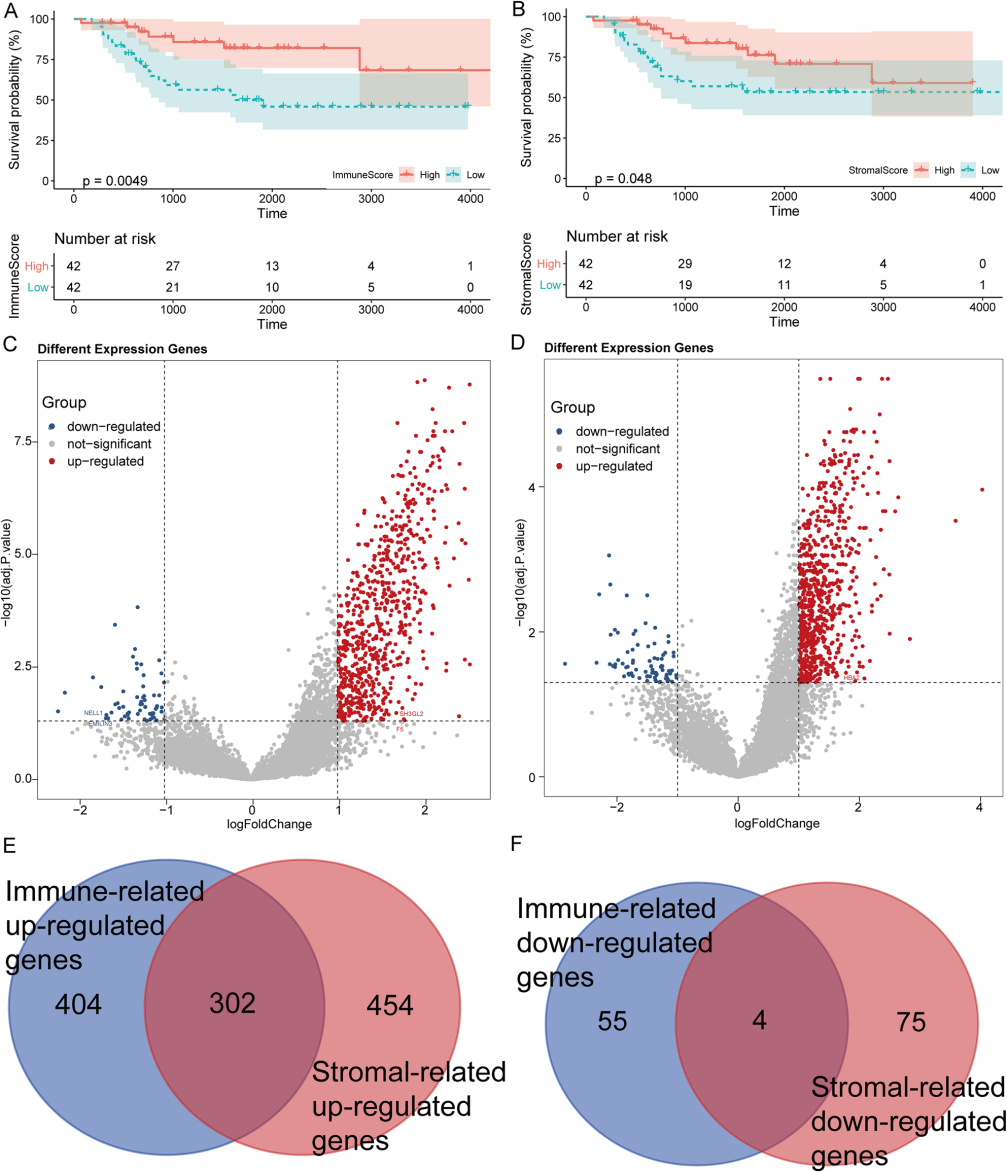
Immune-related DEGs identification. (**A**) Kaplan–Meier survival analysis for osteosarcoma patients grouped into the high or low score in immune score determined by the comparison with the median. p = 0.0049 by log-rank test. (**B**) Kaplan–Meier survival curve for the stromal score with p = 0.048 by log-rank test. (**C**) Volcano plots for DEGs were generated by comparison of the high score group vs. the low score group in the immune score. (**D**) Volcano plots for DEGs were generated by comparison of the high score group vs. the low score group in the stromal score. (**E**,**F**) Venn plots showing common up-regulated and down-regulated DEGs shared by immune score and stromal score.

Patients in the high immune and stromal score group experienced a better prognosis than those in the low immune and stromal score group (Fig. 1A,B). A total of 765 DEGs (705 up-regulated and 60 down-regulated) were identified in the high immune score group (Fig. 1C), whereas 835 DEGs (756 up-regulated and 79 down-regulated) were identified in the high stromal score group (Fig. 1D). The DEGs (302 up-regulated and 4 down-regulated) common to both the groups were selected for further analysis (Fig. 1E,F).

The GO and KEGG enrichment analyses showed the involvement of DEGs in inflammatory responses, including mononuclear and lymphocyte proliferation and activation and T cell activation (Supplementary Fig. S1A,B). The complete GO and KEGG analysis results for all DEGs are shown in Supplementary File 1. In addition, DEGs, including in module 1, were mainly involved in regulating mononuclear and lymphocyte proliferation, macrophage activation, and interleukin-1/10 production (Supplementary File 2). DEGs, including in module 2, were mainly involved in the macrophage activation, cell junction disassembly, and the regulation of cell–cell adhesion (Supplementary File 3). DEGs, including in module 3 (Supplementary File 4) and module 4 (Supplementary File 5), had significant involvement in the immune response, inflammation response, and antigen processing and presentation.

### A three EMT-related genes signature was established

**Figure 2:**
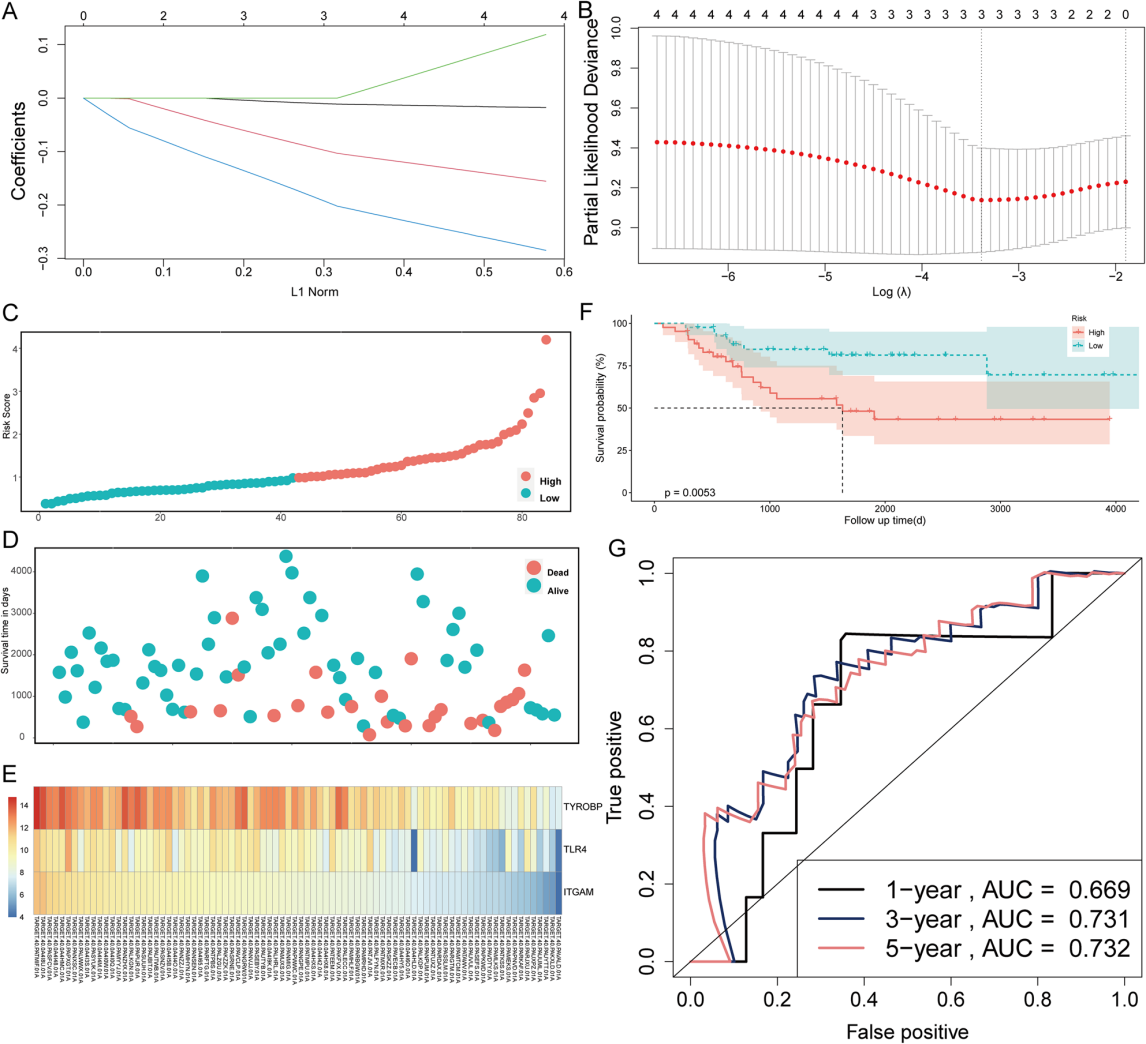
Establishment of a prognostic gene signature for osteosarcoma. (**A**) Using 1000-fold cross-validation to the optimal penalty parameter lambda. (**B**) LASSO coefficient profiles of the 4 DEGs. (**C**) Classification of patients into different risk groups based on the median risk score. (**D**) Distribution of patients’ survival time and status. (**E**) Heatmap of expression profiles of included risk score related genes. (**F**) Kaplan–Meier survival curves between low- and high-risk groups. (**G**) ROC curves of the risk score diagnostic ability.

**Figure 3:**
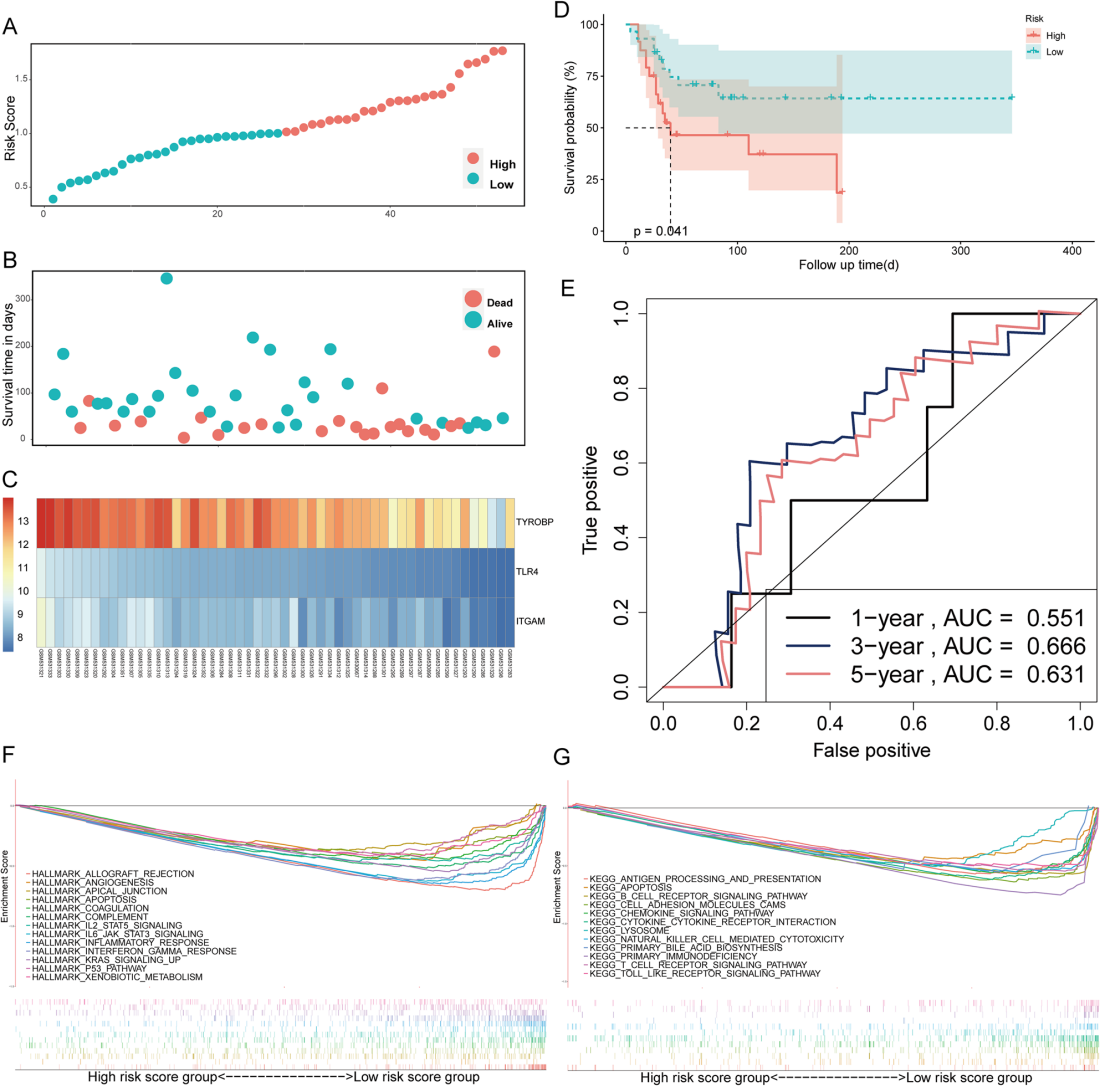
Validation of the prognostic gene signature for osteosarcoma using GSE21257 cohort. (**A**) Classification of patients into different risk groups based on the median risk score. (**B**) Distribution of patients’ survival time and status. (**C**) Heatmap of expression profiles of included risk score related genes. (**D**) Kaplan–Meier survival curves between low- and high-risk groups in the GSE21257 cohort. (**E**) ROC curves of the risk score diagnostic ability. GSEA in the Hallmarks (**F**) and KEGG (**G**) gene set between group high (n = 42) and low (n = 42) of the risk score in the TCGA cohort.

PPI network for all DEGs included 3891 edges and 282 nodes, as shown in Supplementary Fig. S2A. Module 1 (Supplementary Fig. S2B) network consisted of 336 edges and 32 nodes; Module 2 (Supplementary Fig. S2C) network contained 367 edges and 42 nodes; Module 3 (Supplementary Fig. S2D) network consisted of 128 edges and 36 nodes; and Module 4 (Supplementary Fig. S2E) consisted of 51 edges and 28 nodes. We identified TYROBP, TLR4, TLR8, LCP2, ITGAM, LILRB2, and CD86 as hub genes (Supplementary Fig. S3A). We used univariate Cox regression to analyze 7 DEGs and screened 5 DEGs as significant prognosis factors (Table 1). Kaplan–Meier analysis screened 4 DEGs from 7 DEGs affecting OS prognosis (Supplementary Fig. S3SB–H). These four genes, sharing Cox regression analysis and Kaplan–Meier analysis, were further subjected to LASSO regression analysis to select genes included in the risk model (Fig. 2A,B). A 3 EMT-related DEGs (TYROBP, TLR4, and ITGAM) risk signature was established. Patients with continuous risk scores harbored various clinical outcomes in different groups (Fig. 2C–E). Patients in the high-risk group had lower survival rates (Fig. 2F). Moreover, the time-dependent ROC analysis showed that the area under the curve (AUC) value of the three-gene-based model was 0.669, 0.731, and 0.732 at 1, 3, and 5 years, respectively, after diagnosis in the TCGA cohort (Fig. 2G). Similar results were provided by the GSE21257 cohort, which validated the prognostic model (Fig. 3). Furthermore, Supplementary Fig. S4 illustrates that TYROBP, TLR4, and ITGAM were significantly lower in the tumor cells than in the normal bone.

**Table 1:** Univariate Cox regression analyses to the hub genes.

Genes	Full name	HR	95% CI	P value
TYROBP	TYRO protein tyrosine kinase binding protein	0.78	0.64–0.96	0.02056
TLR4	Toll-like receptor 4	0.78	0.64–0.96	0.01886
TLR8	Toll-like receptor 8	0.82	0.66–1	0.05353
LCP2	Lymphocyte cytosolic protein 2	0.75	0.58–0.97	0.02773
ITGAM	Integrin subunit alpha M	0.73	0.58–0.93	0.01058
LILRB2	Leukocyte immunoglobulin like receptor B2	0.81	0.66–0.99	0.03799
CD86	CD86 molecule	0.91	0.7–1.19	0.50322

### Immune-related pathways were enriched in the low-risk group

**Figure 4:**
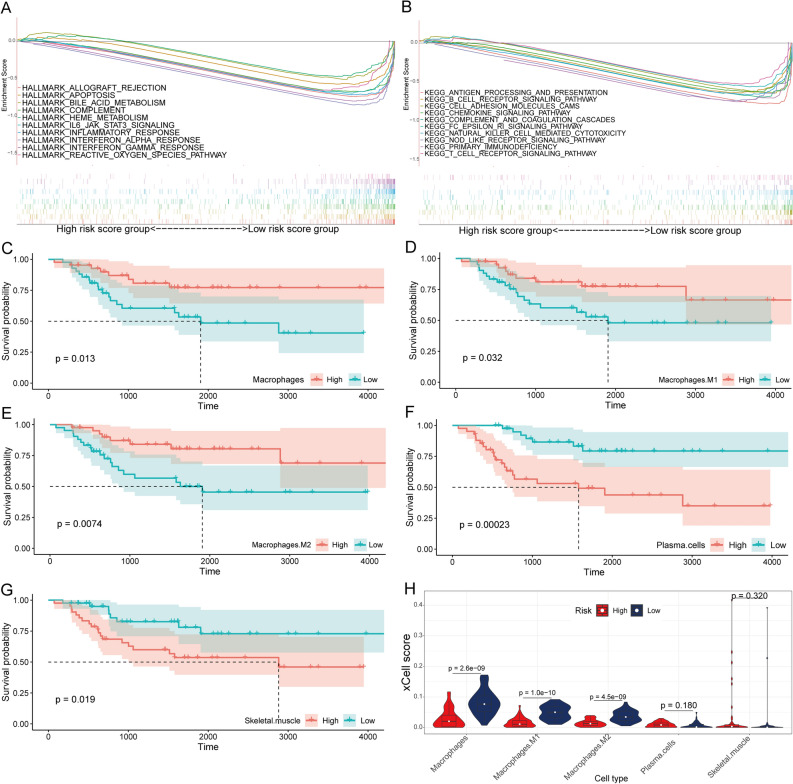
Prognostic-related immune cells identification. GSEA in the Hallmarks (**A**) and KEGG (**B**) gene set between group high (n = 27) and low (n = 26) of the risk score in the GSE21257 cohort. Survival analysis for osteosarcoma patients with different macrophages (**C**), M1 macrophages (**D**), M2 macrophages (**E**), plasma cells (**F**) and skeletal muscle cells (**G**) group. (**H**) Comparison of xCell score of the five prognostic related immune cells between high- and low-risk score group in the TCGA cohort.

The GSEA results for Hallmark and KEGG pathways revealed that most gene sets focused on immune-related pathways. Hallmark results indicated that a majority of the genes were enriched for allograft rejection, IL6/JAK/STAT3 signaling, IL2/STAT5 signaling, and apoptosis (Fig. 3F), whereas, the KEGG background showed enrichment mainly for antigen processing and presentation, Toll-like receptor signaling pathway, cell adhesion molecules (CAMs), and apoptosis (Fig. 3G) in the low-risk group of TCGA cohort. We obtained similar results in the validation cohort (Fig. 4A,B).

### Macrophages were associated with the prognosis and metastatic of OS

**Figure 5:**
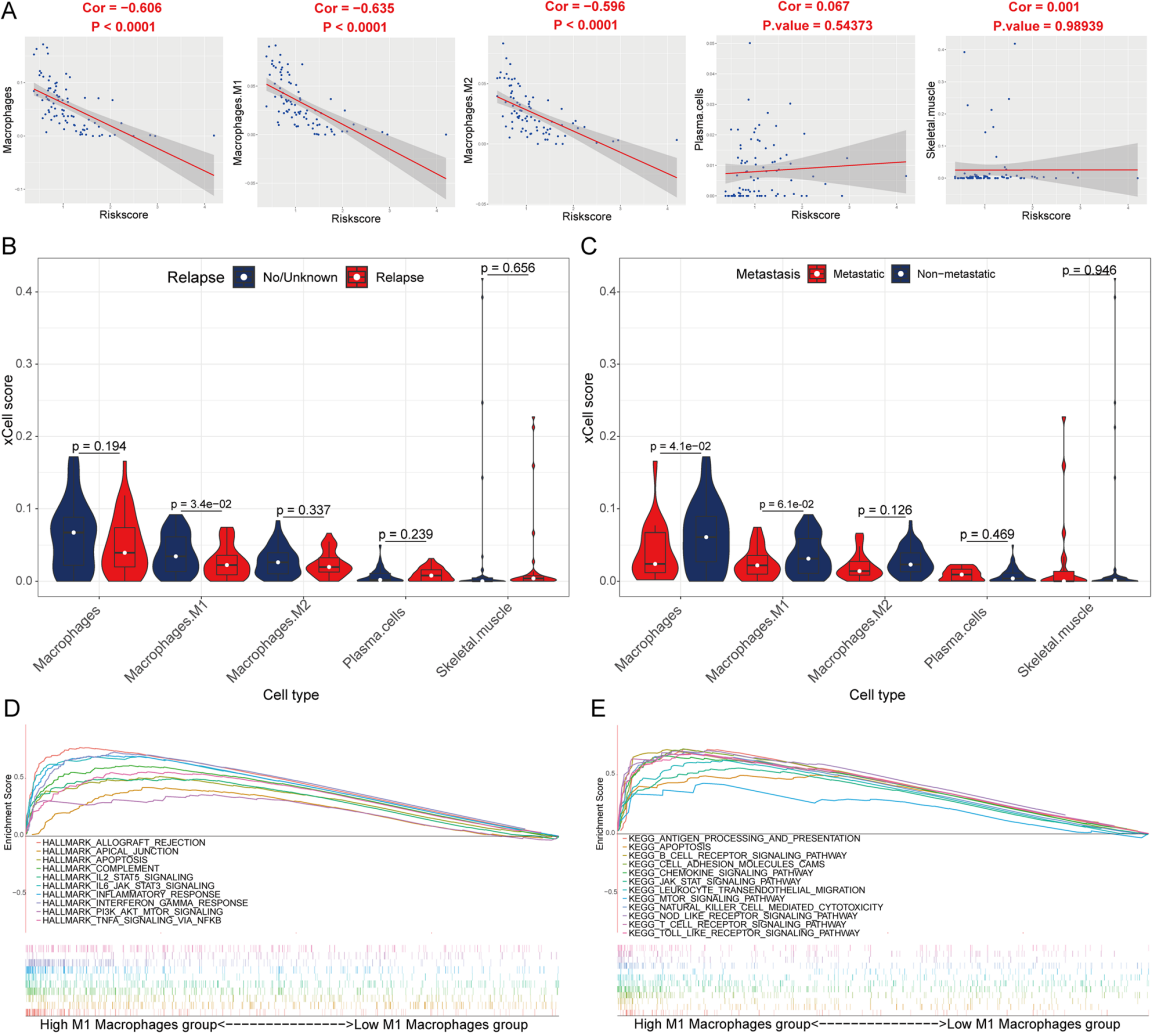
The correlation of risk score with xCell score. (**A**) The correlation between the risk score and xCell score in the TCGA cohort. (**B**,**C**) Comparison of xCell score of the five prognostic related immune cells between relapse/metastatic and non-relapse/non-metastatic group in the TCGA cohort. GSEA in the Hallmarks (**D**) and KEGG (**E**) gene set between group high (n = 42) and low (n = 42) of M1 macrophages in the TCGA cohort.

**Figure 6:**
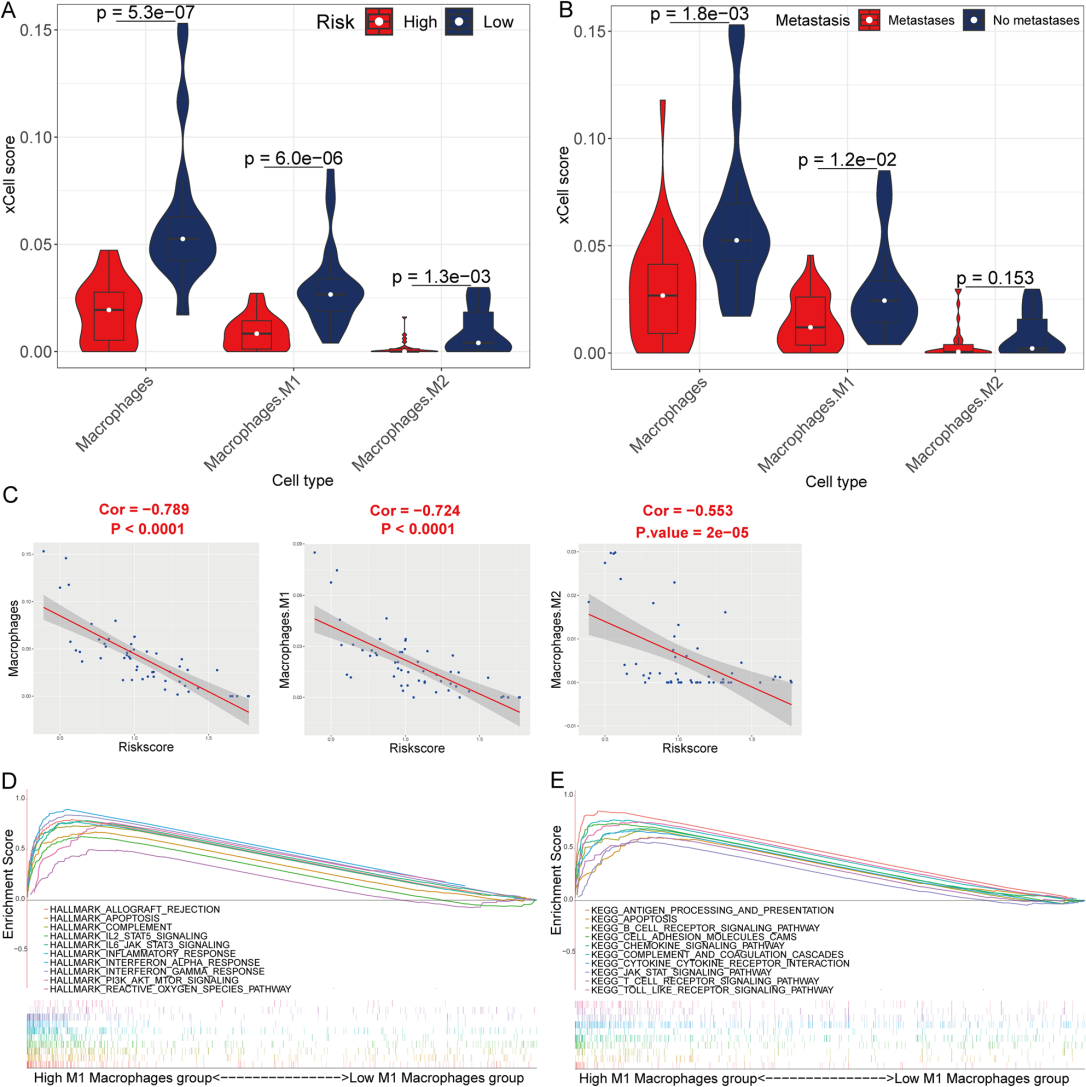
The correlation of risk score with xCell score in the GSE21257 cohort. (**A**) Comparison of xCell score of the five prognostic related immune cells between high- and low-risk score group in the GSE21257 cohort. (**B**) Comparison of xCell score of the five prognostic related immune cells between the metastatic and non-metastatic group in the GSE21257 cohort. (**C**) The correlation between the risk score and xCell score of macrophages, M1 macrophages, M2 macrophages in the GSE21257 cohort. GSEA in the Hallmarks (**D**) and KEGG (**E**) gene set between group high (n = 27) and low (n = 26) of M1 macrophages in the GSE21257 cohort.

Supplementary File 6 shows the xCell score of 64 immune cells of each sample. Macrophages (Fig. 4C), M1 macrophages (Fig. 4D), M2 macrophages (Fig. 4E), plasma cells (Fig. 4F), and skeletal muscle cells (Fig. 4G) had a significant association with OS prognosis. Moreover, macrophages, M1 macrophages, and M2 macrophages were significantly more in the low-risk group than in the high-risk group (Fig. 4H). In addition, macrophages, M1 macrophages, and M2 macrophages negatively correlated with the risk score (Fig. 5A). Macrophages subtypes were significantly more in the non-relapse and non-metastatic groups (Fig. 5B,C). The GSEA results revealed that a majority of the genes showed enrichment for the allograft rejection, IL6/JAK/STAT3 signaling, IL2/STAT5 signaling, and apoptosis (Fig. 5D), whereas, the KEGG background showed enrichment mainly for antigen processing and presentation, Toll-like receptor signaling pathway, CAMs, and apoptosis (Fig. 5E) in the high-M1 macrophages group of TCGA cohort. Figure 6 illustrates similar results for the GSE21257 cohort.

### Risk score potential as an indicator of immunotherapy response in patients with OS

**Figure 7:**
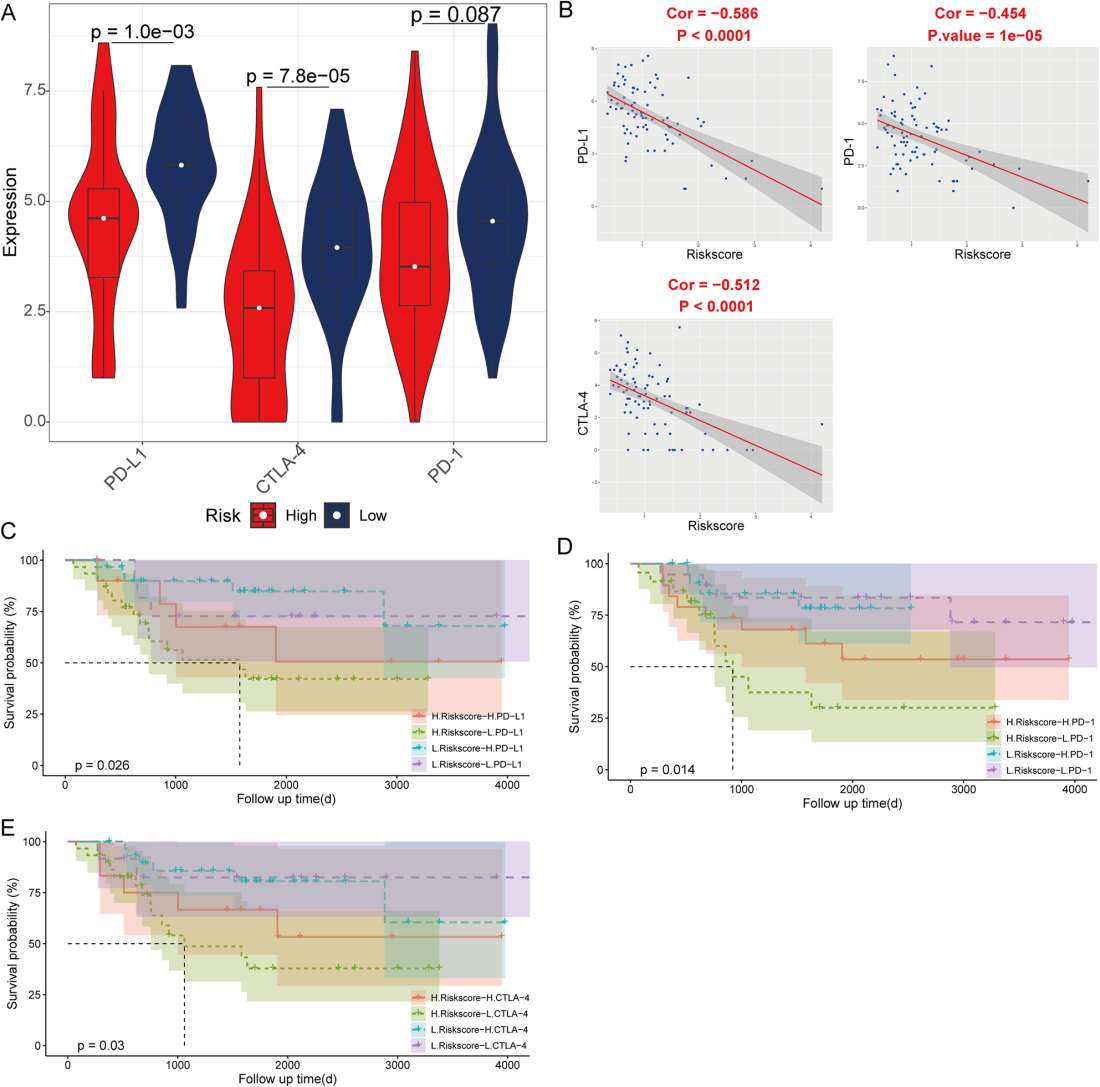
The correlation of risk score with immune checkpoints in the TCGA cohort. (**A**) Comparison of expression of immune checkpoints between high- and low-risk score group (**B**) The correlation between the risk score and expression of immune checkpoints. Kaplan–Meier analysis of the four groups based on the risk score and the expression of PD-L1 (**C**), PD-1 (**D**) and CTLA-4 (**E**).

**Figure 8:**
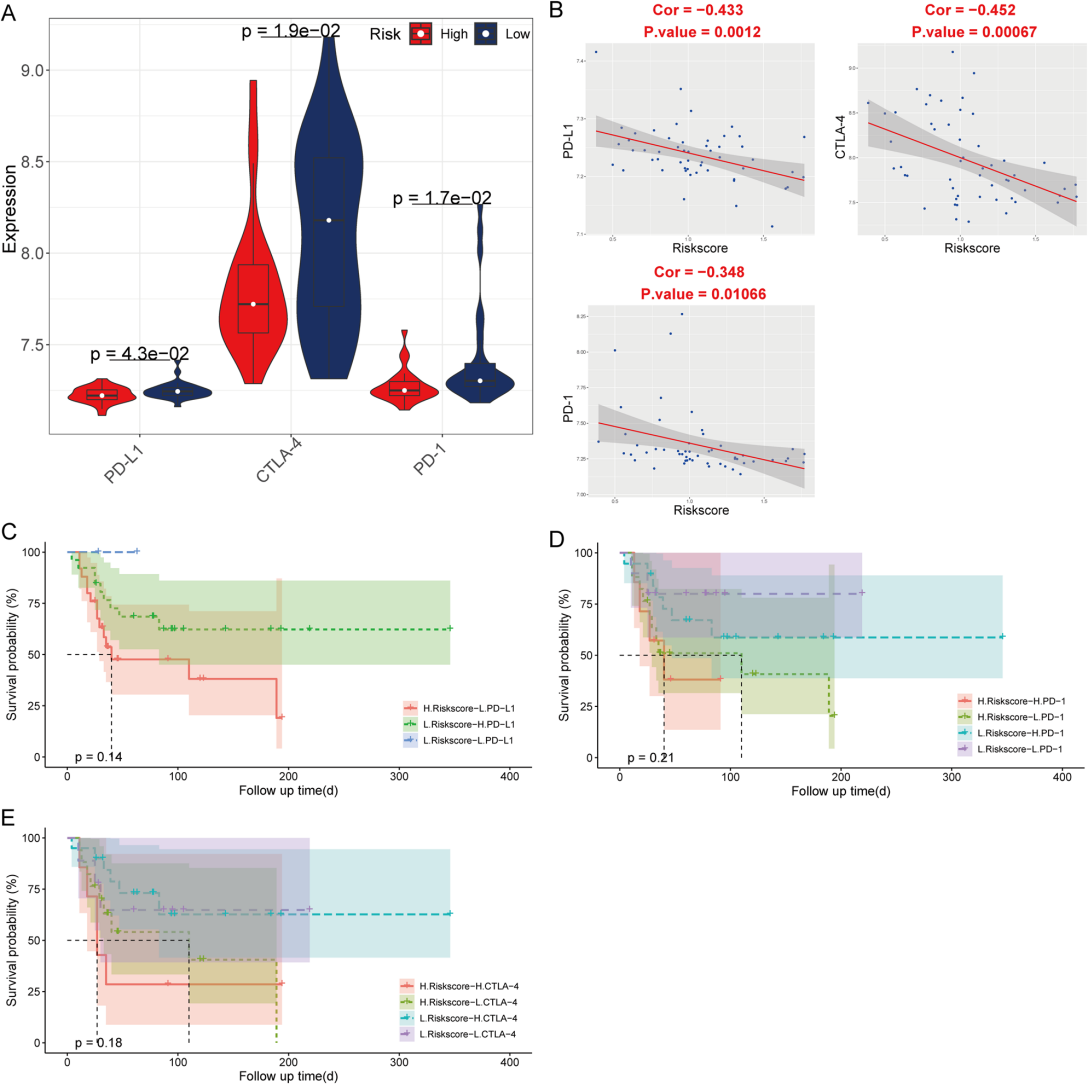
The correlation of risk score with immune checkpoints in the GSE21257 cohort. (**A**) Comparison of expression of immune checkpoints between high- and low-risk score group (**B**) The correlation between the risk score and expression of immune checkpoints. Kaplan–Meier analysis of the four groups based on the risk score and the expression of PD-L1 (**C**), PD-1 (**D**) and CTLA-4 (**E**).

The expression of PD-1, PD-L1, and CTLA-4 was significantly higher in the low-risk score group than in the high-risk score group in the TCGA cohort (Fig. 7A). Immune checkpoint expression negatively correlated with the risk score (Fig. 7B). Patients with low-risk scores may have a better efficacy for immunotherapy in OS (Fig. 7C–E). Moreover, the immune checkpoints expression was also negatively correlated with the risk score in the validation cohort (Fig. 8A,B). The validation cohort showed a better efficacy for immunotherapy in OS (*p* > 0.05) for patients with low-risk scores (Fig. 8C–E).

### Image analysis

**Figure 9:**
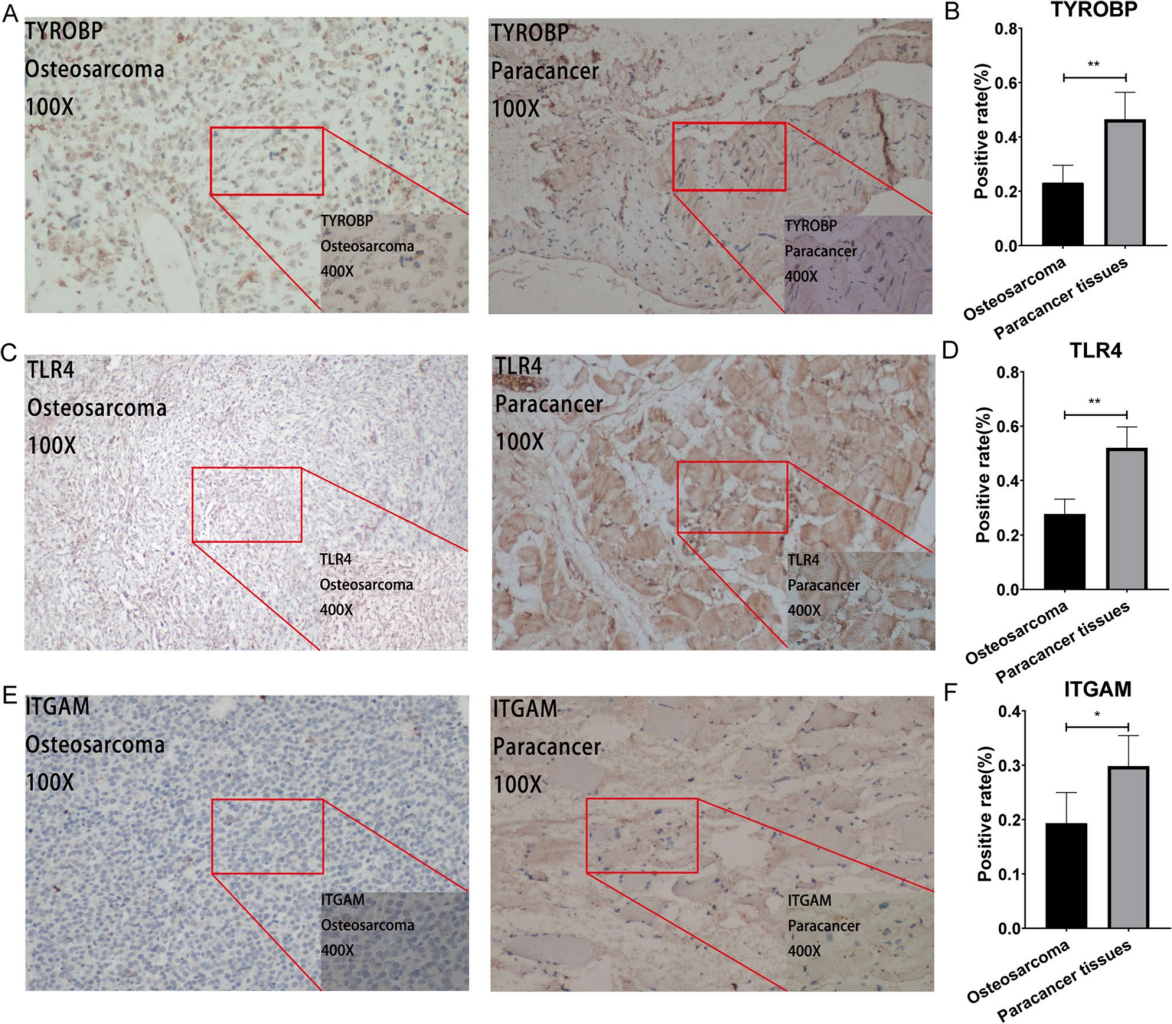
Immunohistochemical plots of the three hub genes associated with prognosis and statistical analysis of the positivity rate. (**A**,**C**,**E**) shows the protein expression of each gene in OS and in the paracancer. (**B**,**D**,**F**) shows the statistical analysis of the staining positivity rate for each gene in OS and in the paracancer. *Representative P-value < 0.05, **representative P-value < 0.01.

All immunohistological images were acquired using an inverted microscope and collected for future use. In addition, we compared the staining of the OS specimens and peritumoral-normal specimens. Representative images in Fig. 9 illustrate that TYROBP, TLR4, and ITGAM were significantly down-regulated in OS than in the peritumoral-normal tissue. In addition, Fig. 9B,D,F showed that a positive rate of immunohistochemical staining for TYROBP, TLR4, and ITGAM in OS, which was significantly lower than that in the peritumoral normal tissue.

## Discussion

The introduced effective therapy has improved in recent years; however, the 5-year survival rates of OS remains low^19,20^. Therefore, searching for effective novel therapies is of utmost importance. Immunotherapy revolutionized the treatment of cancer and has been employed in several cancers. As reported, the DEGs and the TIICs in the TME play a critical role in OS development^21^. Numerous studies have been conducted on OS immunotherapy, such as the TIIC regulation and immune checkpoint blockade^22^. However, studies lack biomarkers to assess patient prognosis and the efficacy of immunotherapy. The purpose of this study was to establish an immune-related gene signature for predicting the prognosis and efficacy of immunotherapy.

We constructed a three-immune-related gene-based risk model to predict the survival rate of OS patients more precisely. Reports state that TYROBP, TLR4, and ITGAM are involved in several cancer-immune microenvironment-associated pathogeneses, including OS^23^. The adapter protein TYROBP non-covalently associates with activating receptors on the surface of various immune cells to mediate signaling and cell activation after ligand binding by the receptors^24^. TYROBP is primarily enriched in natural killer cell-mediated cytotoxicity and osteoclast differentiation, which would lead to tumor cell apoptosis and promote osteoclast differentiation to cause bone resorption around the tumor. According to previous study reports, TYROBP is also involved in the activation of multiple immune cells, including T cells, B cells, and macrophages^24–26^. The low expression of TYROBP may promote the occurrence and progression of OS. As per the risk model, a low expression results in a higher risk.

It has been known that TLR4 plays a fundamental role in pathogen recognition and innate immunity activation^27^. In this study, TLR4 was involved in multiple biological processes (BP), including regulation of cytokine production and secretion, mononuclear cell proliferation, osteoclast differentiation, and macrophage activation. Furthermore, TLR4 activated the immune response in TME by up-regulating multiple immune cells, including T cells, leading to the anti-tumor effect^28–30^. Moreover, TLR4 facilitates osteoclast differentiation that may cause osteolytic destruction in the tumor surrounding area^31,32^. Natural barrier destruction may also be the cause of tumor metastasis. In addition, a high expression of TLR4 may improve prognosis through TME regulation that activates the immune system and facilitates tumor cells apoptosis.

It has been reported that ITGAM is implicated in various adhesive interactions of monocytes, macrophages, and granulocytes, as well as in mediating the uptake of complement-coated particles and pathogens. Studies have reported that ITGAM is significantly associated with tumor metastasis^33,34^. In our study, ITGAM was involved in the cell adhesion molecule pathway and also participated in an important BP, i.e., cell junction disassembly that plays a crucial role in metastasis. Hence, ITGAM may improve the prognosis of OS by inhibiting metastasis.

The literature reports that TYROBP, TLR4, and ITGAM are involved in the BP to activate macrophages that have been reported as tumor-associated and as the main component of the immune environment in OS^35–37^. We found that OS patients with elevated macrophage infiltration in the TME had a better prognosis. Compared to the high-risk score group, patients in the low-risk score group showed a significantly increased number of macrophages, M1 macrophages, and M2 macrophages. Furthermore, compared to non-metastatic/non-relapse cases, the metastatic/relapse cases showed markedly decreased levels of macrophages and M1 macrophages, which satisfactorily suggested that M0 to M1 macrophages polarization levels may be associated with improved outcomes in OS patients. M1 macrophages associated with non-metastasis displayed a pro-inflammatory phenotype and tumoricidal activity in OS^10,37^. Thus, our study results consolidate the previous data based on the beneficial role of M1 macrophage infiltration in OS.

Common immune checkpoint molecules, PD-1, PD-L1, and CTLA-4^38–40^, are known to be related to the OS progress and prognosis. Numerous studies have shown the possibility of immunotherapy in OS^41–43^. Interestingly, the expression of PD‐L1 in OS patients is associated with a higher possibility for obtaining clinical benefits from immunotherapy^44,45^. Our risk score had a significantly negative correlation with PD-1, PD-L1, and CTLA-4. Moreover, TLR4, being an upstream receptor in the PD-L1 expression and PD-1 checkpoint pathway in cancer, was strongly associated with PD-L1 expression. Survival analysis showed that patients with in the low-risk score group experienced higher efficacy in immune checkpoint blockade.

In this study, the specific immune-related gene expression profile-based risk model could more precisely predict the survival of OS patients. Furthermore, the three immune-related genes regulated TIICs in the TME, especially macrophages activation, facilitating the apoptosis of OS cell. Moreover, our study demonstrated that the risk model could predict the immune checkpoint blockade efficacy in OS.

## Conclusion

Overall, a three-immune-related gene-based risk model was constructed that could regulate macrophage activation and predict the survival rate and treatment outcomes to immune checkpoint blockade in OS.

## Patients and methods

### Microarray data acquisition

The TARGET-OS RNA-sequencing dataset (presented as fragments per kilobase million [FPKM]), as well as corresponding clinical characteristics and prognosis information of patients, were downloaded from UCSC Xena (https://xena.ucsc.edu/). However, patients with expression profiles but without clinical characteristics and prognostic information were excluded. Finally, we included 84 patients with OS in the training set. GSE21257 with 53 OS patients was downloaded from the GEO (www. ncbi.nlm.nih.gov/gds), a public repository at the National Center of Biotechnology Information as a validation cohort. In addition, GSE28424 and GSE19276 were downloaded to validate the differential expression of key genes between the tumor cells and normal bone.

### Immune-related DEGs identification and PPI network construction

We applied the “Estimate” package (version 1.0.13) in R language (version 4.0.0) to estimate the immune-stromal component ratio in TME for each sample. TME results consisted of the immune score and stromal score. A higher respective score resulted in a larger ratio of the corresponding component in TME. A total of 84 samples were labeled with either a high score or low score as per the median score obtained for the immune score and stromal score. The “Limma” package (version 3.44.1) was applied to screen DEGs that were defined as an adjusted *p*-value < 0.05 and |log (fold-change)|> 1. Next, we performed the intersection analysis between the up- and down-regulated genes and included only the overlapping genes in the following analysis. The protein–protein interaction (PPI) network was predicted using an online database (STRING; http://string-db.org) (version 11.0b) search tool to retrieve interacting genes and visualized using Cytoscape (version 3.6.1). Molecular Complex Detection (MCODE) is used for clustering a given topology-based network^11^. In addition, clusters with nodes < 20 were discarded.

### GO and KEGG enrichment analysis

All DEGs, including DEGs in each cluster, were analyzed for enrichment using the “clusterProfiler” package **(**version 3.16.0**)** and subsequently visualized using the “ggplot2” package (version 3.3.0) in R language^12^. GO and KEGG^13^ terms with the adjusted *p*-value < 0.05 were considered significantly enriched.

### Hub genes identification and prognostic gene signature established

We used the “cytoHubba” in Cytoscape to identify the hub genes. The top 10 nodes ranked by “Degree,” “Betweenness,” and “Closeness” were enrolled in gene selection. The univariate Cox proportional hazard regression analysis and Kaplan–Meier estimates were used to identify prognostic genes. LASSO regression analyses were used to select the genes included in the model. The cutoff point, Lambda.min, brings minimum mean cross-validated error. We selected the genes with the highest lambda values for further analysis^14^. Next, the risk scores were calculated using the generated coefficients and corresponding expression, and the median risk score was obtained using the prognostic model. Finally, the latter was used to divide the patients into “high-risk” and “low-risk” groups. The diagnostic value of the risk score system was assessed using the time-dependent receiver operating characteristic (ROC) curve and survival analysis.

### Relationships among risk score, immune cells, and immune checkpoints

The xCell tool provides 64 cell types, including lymphoid, myeloid, stromal cells, stem cells, and other cells. Hence, the xCell score analysis using the R package “xCell” (https://github.com/dviraran/xCell) allowed us to obtain 64 immune cell type abundance scores^15^. Furthermore, we performed a Kaplan–Meier analysis to identify prognostic immune cells. The prognostic-immune-cells difference between the two risk groups and clinical characteristics were compared using the *t*-test. The “corrplot” package (version 0.84) was used to perform Pearson’s correlation analysis between the risk score and the xCell score of immunity cells, as well as among the expression of immune checkpoints. Moreover, the prognostic-immune-cells difference between metastatic/relapse and non-metastatic/non-relapse group, and the difference between each immune checkpoint expression in the high- and low-risk score groups were compared using *t*-test. The patients were grouped into four groups based on the risk score and immune checkpoint expression. Lastly, their relationships with overall survival were analyzed using Kaplan–Meier analysis. We considered a *p*-value < 0.05 as the threshold for significance.

### Gene set enrichment analysis (GSEA)

The computational method, GSEA (V4.0.3), determines whether a priori defined set of genes shows statistically significant concordant differences between two biological states^16^. Herein, GSEA investigated the potential biological characteristics between different statuses based on gene sets downloaded from the Molecular Signatures Database^17^. In this study, we only investigated the Hallmarks (h.all.v72.symbols.gmt) and c2 (c2.cp.kegg.v7.2.symbols.gmt) gene sets. The significant cutoff value was defined as the false discovery rate (FDR) < 0.25 and the normal *p* < 0.05.

### Immunohistochemistry

The subjects volunteering for this study had signed informed consent forms. Moreover, our study was approved by the Ethics Department of the First Affiliated Hospital of Guangxi Medical University and conformed to the World Medical Association Declaration of Helsinki. Six pairs (OS and peritumoral normal tissue) of pathological sections for each gene were immunohistologically analyzed. For immunohistochemical staining, the TYROBP, TLR4 and ITGAM antibodies were purchased from Abcam. The paraffin, hydration, and seals were removed. Next, the specimens were mixed with anti-TYROBP, TLR4, and ITGAM antibodies at various dilution ratios (1:250, 1:200, 1:4000, 1:300, and 1:100) and incubated overnight at 4 °C^18^. Lastly, we statistically analyzed the positivity rate of the immunohistology images using Image J software and GraphPad Prism 8.

### Ethics approval and consent to participate

All subjects volunteered for the study and signed informed consent forms. In order to ensure confidentiality, the names of study participants were not included in the data. Information obtained from the data of the study participants is kept confidential. In addition, the Ethics Committee of The First Affiliated Hospital of Guangxi Medical University approved the study.

### Consent to publish

Consent for publication was obtained from all participants.

## Supplementary Information

Supplementary Figure 1.

Supplementary Figure 2.

Supplementary Figure 3.

Supplementary Figure 4.

Supplementary Information 1.

Supplementary Information 2.

Supplementary Information 3.

Supplementary Information 4.

Supplementary Information 5.

Supplementary Information 6.

Supplementary Legends.

## Data Availability

The dataset generated or analyzed during the current study are available in the TCGA dataset repository (https://tcga-data.nci.nih.gov/tcga/) and Gene Expression Omnibus (GEO, https://www.ncbi.nlm.nih.gov/geo/). And all data and materials for this study shall be availed whenever requested by the editorial team, reviewers, and other users.

## Abbreviations

GSEA: Gene Set Enrichment Analysis
GO: Gene ontology
KEGG: Kyoto Encyclopedia of Genes and Genomes
DEGs: Differentially expressed genes
TYROBP: TYRO protein tyrosine kinase binding protein
TLR4: Toll-like receptor 4
ITGAM: Integrin subunit alpha M
PD-1: Programmed Cell Death 1
PD-L1: Programmed Cell Death 1 Ligand 1
CTLA-4: Cytotoxic T-Lymphocyte Associated Protein 4
TCGA: The Cancer Genome Atlas Program
TME: Tumor microenvironment
TIICs: Tumor-infiltrating immune cells
DEGs: Differentially expressed genes
