# Supplementary figures and images for: TYROBP, TLR4 and ITGAM regulated macrophages polarization and immune checkpoints expression in osteosarcoma
Source: Sci Rep. 2021 Sep 29;11:19315. doi: 10.1038/s41598-021-98637-x (PMC8481262; doi:10.1038/s41598-021-98637-x)

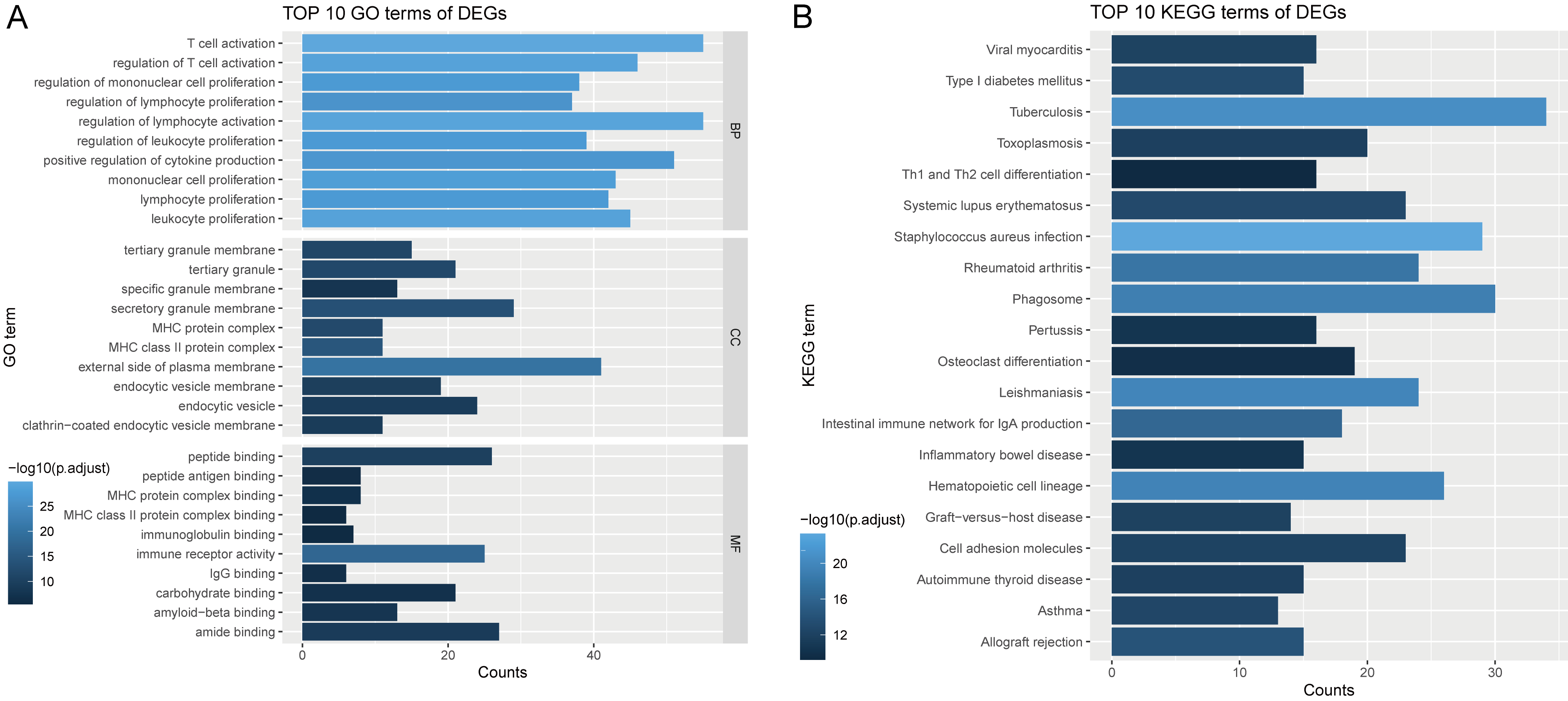

Supplement: Supplementary file 1 — Supplementary Figure 1. [file 41598_2021_98637_MOESM1_ESM.tif]

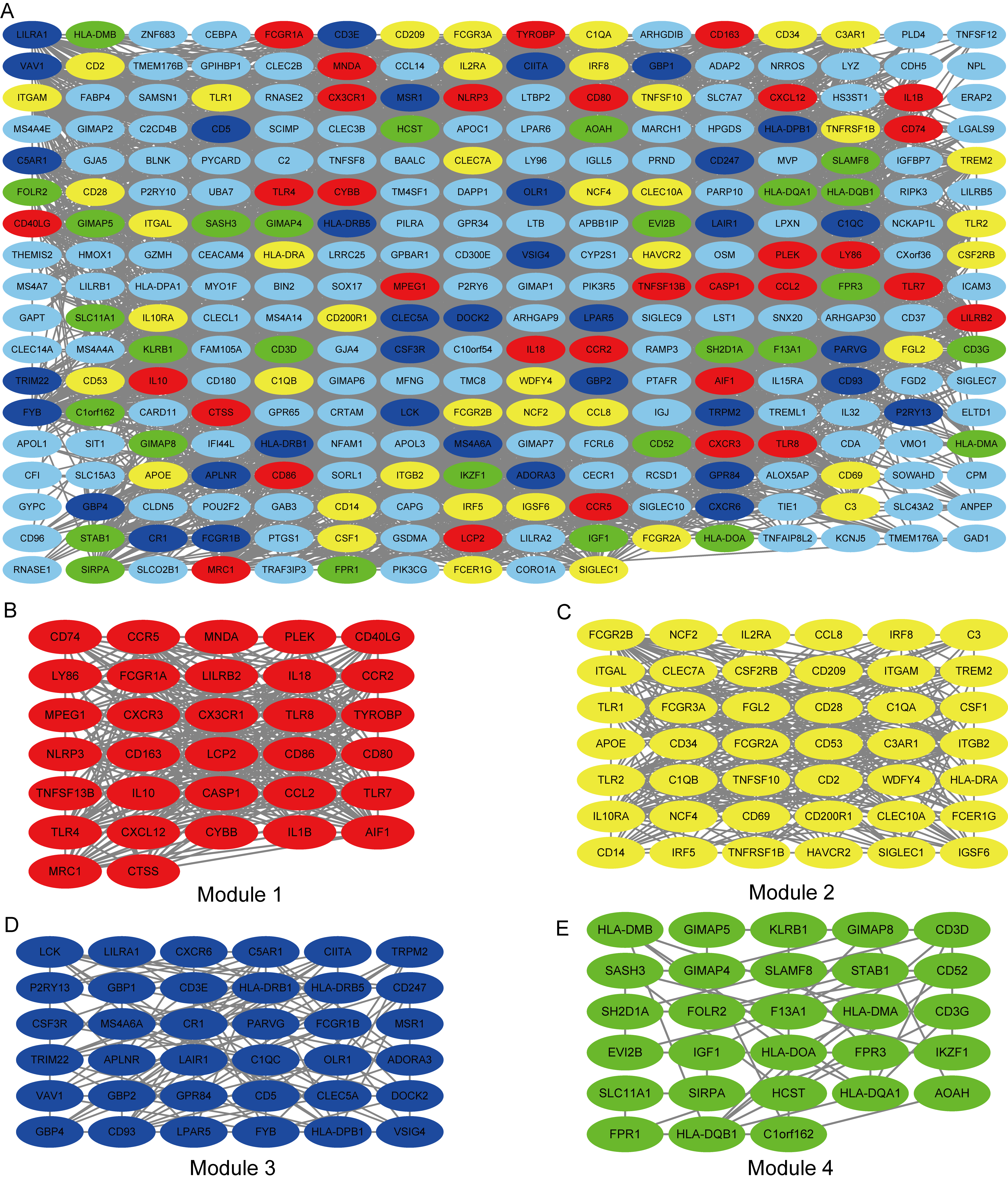

Supplement: Supplementary file 2 — Supplementary Figure 2. [file 41598_2021_98637_MOESM2_ESM.tif]

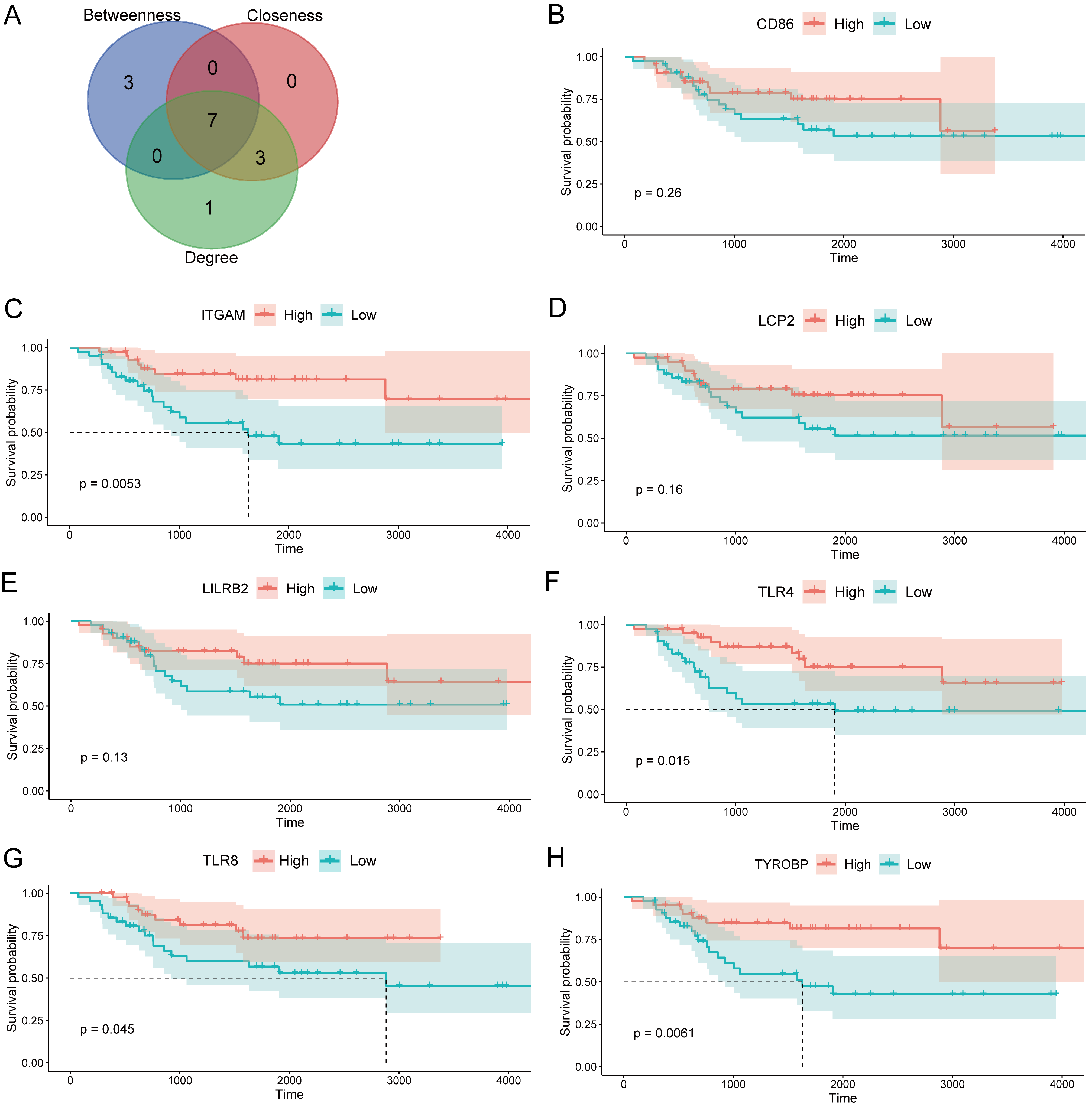

Supplement: Supplementary file 3 — Supplementary Figure 3. [file 41598_2021_98637_MOESM3_ESM.tif]

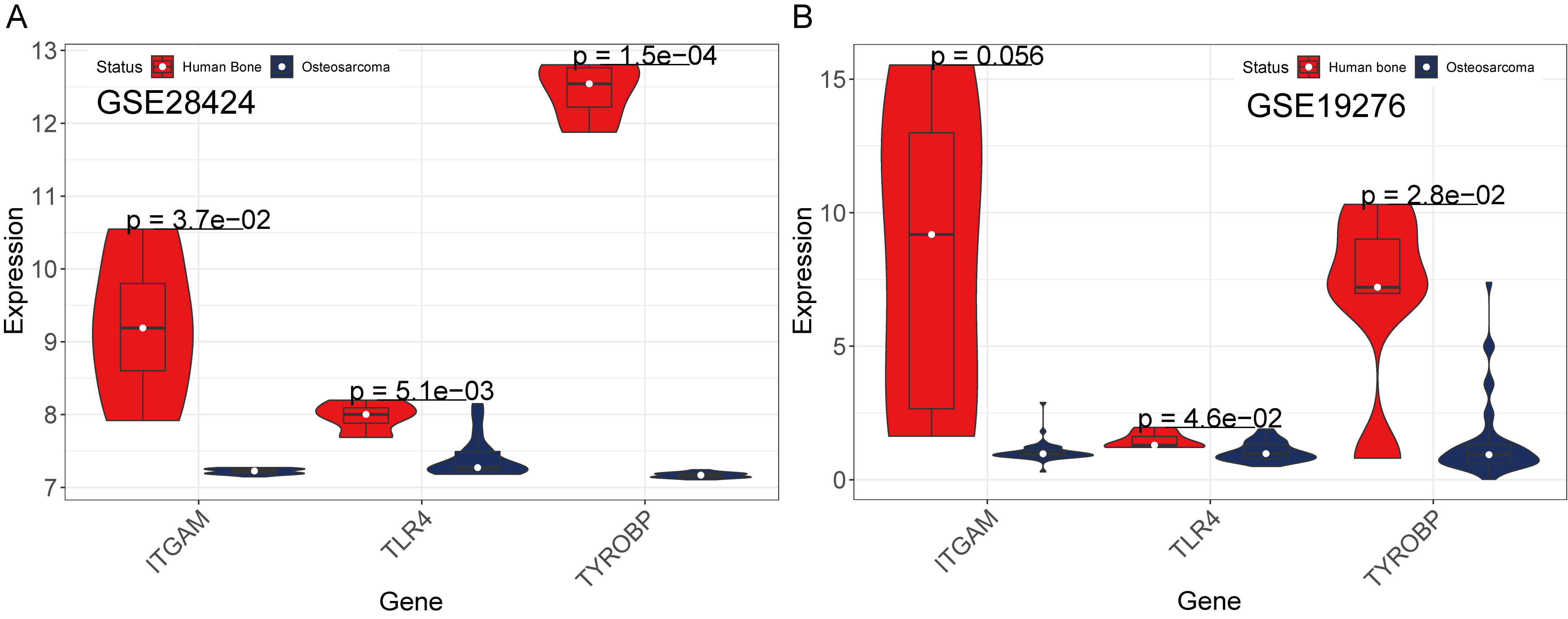

Supplement: Supplementary file 4 — Supplementary Figure 4. [file 41598_2021_98637_MOESM4_ESM.tif]
