# Supplementary material for: TYROBP, TLR4 and ITGAM regulated macrophages polarization and immune checkpoints expression in osteosarcoma
Source: Sci Rep. 2021 Sep 29;11:19315. doi: 10.1038/s41598-021-98637-x (PMC8481262; doi:10.1038/s41598-021-98637-x)
Supplement: Supplementary file 11 — Supplementary Legends. [file 41598_2021_98637_MOESM11_ESM.pdf]

TYROBP, TLR4 and ITGAM Regulated Macrophages Polarization and Immune Checkpoints Expression in Osteosarcoma

Tuo Liang1, Jiarui Chen1, GuoYong Xu1, Zide Zhang1, Jiang Xue1, Haopeng Zeng1, Jie Jiang1, Tianyou Chen1, Zhaojie Qin1, Hao Li1, Zhen Ye1, Yunfeng Nie2, Chong Liu1*, Xinli Zhan1*

1 Department of Spine and Osteopathy Ward, The First Affiliated Hospital of Guangxi Medical University, No. 6 Shuangyong Road, Nanning, Guangxi, P. R. China. Fax: +86 771 5350021;

2 Guangxi Medical University, No.22 Shuangyong Road, Nanning, Guangxi, People's Republic of China. Fax: +86 771 5350021;

*Corresponding author at Xinli Zhan, Department of Spine and Osteopathy Ward, The First Affiliated Hospital of Guangxi Medical University, No. 6 Shuangyong Road, Nanning, Guangxi 530021, P. R. China (e-mail: zhanxinli@stu.gxmu.edu.cn).

Supplemental legends:

Supplemental Figure 1. Enrichment analysis of GO and KEGG for DEGs. (A) GO enrichment analysis for all DEGs. (B) KEGG enrichment analysis for all DEGs.

Supplemental Figure 2. The PPI network analysis of immune DEGs in osteosarcoma. (A) The PPI network analysis using STRING online tools and visualization by Cytoscape 3.7.1 software. Interacting genes with the highest MCODE scores were clustered in module 1, which is indicated as red nodes. Modules 2–6 are shown in yellow, blue and green, respectively. Interacting genes in module 1 (B), module 2 (C), module 3 (D), module 4 (D).

Supplemental Figure 3. Venn plots and prognostic value of overlap genes in osteosarcoma by Kaplan–Meier analysis. (A) Venn plot showing the hub genes shared by degree, closeness and betweenness. Survival analysis for osteosarcoma patients with

different CD86 (B), ITGAM (C), LCP2 (D), LILRB2 (E), TLR4 (F), TLR8 (G) and TYROBP (H) expression.

Supplemental Figure 4. Genes expression validation. ITGAM, TLR4 and TYROBP between normal bone and osteosarcoma in GSE28424 (A) and GSE19276 (B).

Supplemental files 1: GO and KEGG enrichment analysis for all DEGs.

Supplemental files 2: GO and KEGG enrichment analysis for DEGs in cluster 1.

Supplemental files 3: GO and KEGG enrichment analysis for DEGs in cluster 2.

Supplemental files 4: GO and KEGG enrichment analysis for DEGs in cluster 3.

Supplemental files 5: GO and KEGG enrichment analysis for DEGs in cluster 4.

Supplemental files 6: xCell score of 64 subtypes immune cells of each sample.
